# 10 Years of Pressurized Intraperitoneal Aerosol Chemotherapy (PIPAC): A Systematic Review and Meta-Analysis

**DOI:** 10.3390/cancers15041125

**Published:** 2023-02-09

**Authors:** Andrea Di Giorgio, Antonio Macrì, Federica Ferracci, Manuela Robella, Mario Visaloco, Giovanni De Manzoni, Paolo Sammartino, Antonio Sommariva, Daniele Biacchi, Franco Roviello, Roberta Pastorino, Denise Pires Marafon, Stefano Rotolo, Francesco Casella, Marco Vaira

**Affiliations:** 1 Surgical Unit of Peritoneum and Retroperitoneum, Fondazione Policlinico Universitario A. Gemelli—IRCCS, 00168 Rome, Italy; 2 U.O.C.—P.S.G. con O.B.I. Azienda Ospedaliera Universitaria “G. Martino”—Messina, 98125 Messina, Italy; 3 Candiolo Cancer Institute, FPO—IRCCS, Candiolo, 10060 Torino, Italy; 4 Upper GI Surgery Division, University of Verona, 37129 Verona, Italy; 5 CRS and HIPEC Unit, Pietro Valdoni, Umberto I Policlinico di Roma, 00161 Roma, Italy; 6 Advanced Surgical Oncology Unit, Surgical Oncology of the Esophagus and Digestive Tract, Veneto Institute of Oncology IOV-IRCCS, 35128 Padova, Italy; 7 Department of Medicine, Surgery, and Neurosciences, Unit of General Surgery and Surgical Oncology, University of Siena, 53100 Siena, Italy; 8 Sezione di Igiene, Dipartimento Universitario Scienze della Vita e Sanità Pubblica, Università Cattolica del Sacro Cuore, 00168 Roma, Italy; 9 Department of Woman and Child Health and Public Health—Public Health Area, Fondazione Policlinico Universitario A. Gemelli—IRCCS, 00168 Roma, Italy; 10 Department of Surgical, Oncological and Oral Sciences (Di.Chir.On.S.), University of Palermo, 90133 Palermo, Italy

**Keywords:** pressurized intraperitoneal aerosol chemotherapy (PIPAC), peritoneal metastases, carcinomatosis, aerosol chemotherapy, locoregional chemotherapy, neoadjuvant treatment, response assessment

## Abstract

**Simple Summary:**

In recent years, pressurized intraperitoneal aerosol chemotherapy (PIPAC) has emerged as a feasible method of intraperitoneal drug administration in patients affected by peritoneal cancer of primary or secondary origin. We performed a systematic review and meta-analysis with the aim of assessing the feasibility, safety, and efficacy of PIPAC.

**Abstract:**

Background: Pressurized intraperitoneal aerosol chemotherapy (PIPAC) is a novel intraperitoneal drug delivery method of low-dose chemotherapy as a pressurized aerosol in patients affected by peritoneal cancer of primary or secondary origin. We performed a systematic review and meta-analysis with the aim of assessing the feasibility, safety, and efficacy of PIPAC. Methods: A systematic literature search was performed using Medline and Web of Science databases from 1 January 2011, to inception, to 31 December 2021. Data were independently extracted by two authors. The Newcastle-Ottawa Scale was used to assess the quality and risk of bias of studies. Meta-analysis was performed for pathological response, radiological response, PCI variation along treatment, and for patients undergoing three or more PIPAC. Pooled analyses were performed using the Freeman–Tukey double arcsine transformation, and 95% CIs were calculated using Clopper–Pearson exact CIs in all instances. Results: A total of 414 papers on PIPAC were identified, and 53 studies considering 4719 PIPAC procedure in 1990 patients were included for analysis. The non-access rate or inability to perform PIPAC pooled rate was 4% of the procedures performed. The overall proportion of patients who completed 3 or more cycles of PIPAC was 39%. Severe toxicities considering CTCAE 3–4 were 4% (0% to 38.5%). In total, 50 studies evaluated deaths within the first 30 postoperative days. In the included 1936 patients were registered 26 deaths (1.3%). The pooled analysis of all the studies reporting a pathological response was 68% (95% CI 0.61–0.73), with an acceptable heterogeneity (I^2^ 28.41%, *p* = 0.09). In total, 10 papers reported data regarding the radiological response, with high heterogeneity and a weighted means of 15% (0% to 77.8%). PCI variation along PIPAC cycles were reported in 14 studies. PCI diminished, increased, or remained stable in eight, one and five studies, respectively, with high heterogeneity at pooled analysis. Regarding survival, there was high heterogeneity. The 12-month estimated survival from first PIPAC for colorectal cancer, gastric cancer, gynecological cancer and hepatobiliary/pancreatic cancer were, respectively, 53%, 25%, 59% and 37%. Conclusions: PIPAC may be a useful treatment option for selected patients with PM, with acceptable grade 3 and 4 toxicity and promising survival benefit. Meta-analysis showed high heterogeneity of data among up-to-date available studies. In a subset analysis per primary tumor origin, pathological tumor regression was documented in 68% of the studies with acceptable heterogeneity. Pathological regression seems, therefore, a reliable outcome for PIPAC activity and a potential surrogate endpoint of treatment response. We recommend uniform selection criteria for patients entering a PIPAC program and highlight the urgent need to standardize items for PIPAC reports and datasets.

## 1. Introduction

Pressurized intraperitoneal aerosol chemotherapy (PIPAC) is a novel intraperitoneal drug delivery method of low-dose chemotherapy as a pressurized aerosol in patients affected by peritoneal cancer of primary or secondary origin.

PIPAC combines the theoretical pharmacokinetic advantages of low-dose intraperitoneal chemotherapy (i.e., low toxicity, high intraperitoneal concentration, and low systemic concentration) with the principles of the aerosol (homogeneous intraperitoneal distribution and deeper tissue penetration).

Currently available treatments, consisting of palliative systemic chemotherapy, are poorly effective against peritoneal metastases, determining minimal clinical benefit and poor survival results.

In recent years, many studies have shown that PIPAC is a feasible method for intraperitoneal drug administration and, given its minimally invasive nature and safety profile, seems a promising tool for implementing a more comprehensive treatment of patients suffering from peritoneal metastases (PM) [1].

We performed a systematic review and meta-analysis with the aim of assessing the feasibility, safety, and efficacy of PIPAC.

## 2. Materials and Methods

This systematic review and meta-analysis was performed according to the Cochrane Handbook for systematic reviews and to the Preferred Reporting Items for Systematic Reviews and Meta-Analyses (PRISMA) statement; the PRISMA checklist is available as Supplementary Material (Table S1) [2,3]. It was registered in the International Prospective Register of Systematic Reviews PROSPERO [4] as CRD42022320389 [5].

### 2.1. Electronic Searches and Selection Criteria

The search was performed by two investigators (FF and ADG) independently using two different electronic databases: Medical Literature Analysis and Retrieval System Online (MEDLINE) via PubMed and ISI Web of Science (WOS).

The comprehensive string search for each database is shown in Table 1.

The search was limited to studies published between 1 January 2011 (the year PIPAC was first used in humans) [6] and 31 December 2021.

All articles generated from the electronic search were imported into Mendeley© (Elsevier, Amsterdam, The Netherlands), a reference management software, and duplicates were removed.

The study selection process was independently carried out by four reviewers (FF, ADG, MR, and AM).

Papers were eligible if they included at least two patients with PM treated with PIPAC, with or without concomitant systemic chemotherapy. The eligible study designs included randomized controlled trials, case series, and retrospective/prospective cohorts of patients.

Studies that performed in vitro or in animal research, studies on environmental safety, meeting abstracts, comments, correspondence letters, editorials, narrative reviews, case reports, and studies written in a non-English language were not considered eligible.

Differences in judgment during the selection process between the four reviewers were resolved by discussion and consensus.

### 2.2. Aims

The main aims of the present review and meta-analysis were to assess the feasibility, safety, and efficacy of PIPAC:

The feasibility evaluation was based on the rate of non-access to the abdominal cavity and technical issues impeding PIPAC administration. The proportion of patients who completed three or more cycles of PIPAC was also reported as a secondary measure of feasibility.

The safety was evaluated on severe adverse events, according to the Common Terminology Criteria for Adverse Events (CTCAE 3–4).

The efficacy was studied in terms of pathological response rate (calculated as the number of patients that showed any histological response according to the PRGS or TRG score (or other systems) scores to PIPAC treatment on the total number of patients evaluated for this outcome in each study). Macroscopic response according to Peritoneal Cancer Index (PCI) and radiological response according to RECIST criteria v.1.1 were also evaluated.

Finally, a survival analysis in terms of estimated overall survival was attempted.

### 2.3. Data Extraction

Information was collected using an Excel© (Microsoft Office, Redmond, WA, USA) spreadsheet specifically developed for this study, including first author’s name; year of publication; study design; sample size; the number of patients treated and number of PIPAC cycles administered; origin of primitive tumor; previous systemic chemotherapy; combined systemic chemotherapy; access failure; PCI at first PIPAC; performance status (PS) at first PIPAC according to the Eastern Cooperative Oncology Group scale (ECOG); ascites at first PIPAC; severe toxicity events according to the CTCAE v. 4.0; pathological responses, including complete, major, minor response using the PRGS, TRG, or other systems; radiological objective response rate, including complete and partial response according to the RECIST criteria v1.1; and overall survival. Severe toxicity was calculated as the events grade 3–4 according to CTCAE on the total number of PIPAC procedures.

### 2.4. Risk of Bias and Quality Assessment

We performed the quality assessment of the included studies by using the Newcastle-Ottawa quality assessment scale. This scale varies from 0 (lowest quality score) to a maximum possible score of 9 (highest quality score) and incorporates information on participant selection, outcome and exposure ascertainment, and the potential for confounding.

### 2.5. Data Synthesis and Statistical Analysis

The main results of the systematic review were displayed on a finding table, with descriptively pooled outcome data (weighted means) according to tumor origin.

For each study, a description of the population, outcomes measures, and results were presented.

Mean and standard deviation (SD) or median and range were collected depending on the data available in the papers.

All analyses were performed in Stata/SE 16 using the metaprop command to calculate pooled proportions. Meta-analysis was performed by using as key outcomes the pathological response, the radiological response, the PCI variation along PIPAC treatment, and the number of patients undergoing three or more PIPAC cycles. In order to reduce the heterogeneity, a sub-analysis per tumor origin, excluding those studies with PM from various entity, was caried out for the pathological response and for the number of patients undergoing three or more PIPAC cycles. Freeman–Tukey double arcsine transformation was used, and 95% CIs were calculated using Clopper–Pearson exact CIs in all instances.

Heterogeneity between studies was estimated using the I^2^ statistic; because of the heterogeneity of the included studies, all analyses used inverse-variance weighted random-effects models using the DerSimonian–Laird method.

A random-effects metaregression was applied to evaluate the influence of continuous covariates (previous systemic chemotherapy, concomitant systemic chemotherapy, ECOG PS at first PIPAC, ascites at first PIPAC and CTCAE ¾) on the key outcomes, with *p*-values calculated using a Monte Carlo permutation test.

Concerning the survival analysis, the survival rates at 3, 6, 9, and 12 months were estimated with the function S(t) = e^−ht^, where t is the time and h is the HR (hazard ratio) calculated from the median survival time (MST) (h = ln(2)/MST) [7]. Survival was estimated on studies with PM from a unique primary malignancy.

## 3. Results

### 3.1. Study Selection

**Figure 1:**
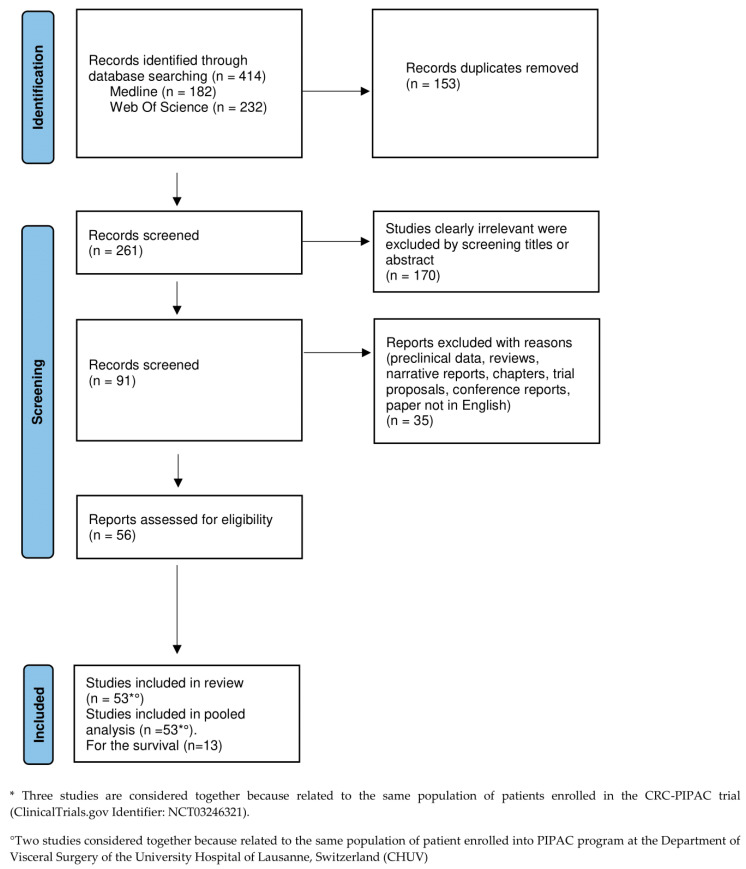
Study selection process (PRISMA flow diagram).

The study selection process and the results of the literature search are shown in Figure 1. In total, 414 studies were identified from the two databases (182 from Medline and 232 from Web of Science), of which 53 were finally included in the systematic review process [8,9,10,11,12,13,14,15,16,17,18,19,20,21,22,23,24,25,26,27,28,29,30,31,32,33,34,35,36,37,38,39,40,41,42,43,44,45,46,47,48,49,50,51,52,53,54,55,56,57,58,59,60]. 

Lurvink et al., 2020; Lurvink et al., 2021; and Rovers et al., 2021 were considered as one study since they report the data of the same population enrolled in the CRC-PIPAC trial (ClinicalTrials.gov Identifier: NCT03246321) [39,61,62].

Similarly, Hubner et al., 2017 and Teixeira Farinha et al., 2017 provided results concerning the same population of 42 patients included in their PIPAC program and were considered as 1 [25,63].

Definitively, a total of 1990 patients and 4719 PIPAC procedures were involved in the systematic review.

### 3.2. Quality Assessment

NOS was used to assess the quality of the study included, which ranged from 3 to 9 stars. One study (1.9%) was scored 9, 10 (18.9%) were scored 8, 17 (32%) were scored 7, 16 (30.2%) were scored 6, 5 (9.4%) were scored 5, 3 (5.7%) were scored 4, and 1 (1.9%) was scored 3. The table is available as Supplementary Material (Table S2).

### 3.3. Study Characteristics

Within 2014 and 2021, 4 phase I and 5 phase II trials were published. Indeed, most of the included studies were prospective or retrospective cohort studies (*n* = 44), including 2 case series. There were no phase III studies or randomized trials reported at the time of the search. Concerning phase I studies, summarized in Table 2, Tempfer et al., 2018 was the first dose escalation study for PIPAC-Doxorubicin/Cisplatin and was terminated without dose-limiting toxicity (DLT) and without reaching the maximally tolerated dose (MTD), suggesting an increase in the dose of the drugs compared to the previously used dosages (Doxorubicin 2.1 mg/m^2^—Cisplatin 10.5 mg/m^2^ vs. Doxorubicin 1.5 mg/m^2^—Cisplatin 7.5 mg/m^2^). Dumont et al., 2020 and Kim et al., 2021 reported the results of two dose-escalation studies for PIPAC-Oxaliplatin, conducted in the same time period. The first study was terminated at a dose of 90 mg/m^2^ due to DLTs, and the second was terminated with the final escalation dose of 120 mg/m^2^, without reaching the MTD. Robella et al., 2021 suggested 135 mg/m^2^ for PIPAC-Oxaliplatin and for PIPAC-Cisplatin/Doxorubicin 6 mg/m^2^ and 30 mg/m^2^, significantly higher doses without DLTs and without reaching the MTD. To be thorough, we must mention a phase I trial recently published beyond the term of our research by Ceelen et al., 2022, the PIPAC Nabpaclitaxel dose-escalation study [64].

The phase II trials enrolled patients with PM from a single origin (Rovers et al., 2021 colorectal cancer; Khomyakov et al., 2016 and Struller et al., 2019 gastric cancer; and Tempfer et al., 2015 ovarian cancer), except De Simone et al., 2020, which involved patients with PM from various origin. In all studies were used Oxaliplatin at 92 mg/m^2^ for PM from colorectal origin and Cisplatin 7.5 mg/m^2^—Doxorubicin 1.5 mg/m^2^ for ovarian, gastric, and primary peritoneal cancers. All studies but Khomyakov et al., 2016 evaluated the efficacy of PIPAC treatment in terms of radiological response according to RECIST criteria v 1.1. Including the stable disease in their primary endpoint, Struller et al., 2019, Tempfer et al., 2015, and De Simone et al., 2020 observed a radiological response rate of 40%, 62%, and 35% respectively. Differently, Rovers et al., 2021 did not consider the stable disease, and no patient reached the endpoint. Instead, Khomyakov et al., 2016 evaluated the efficacy of the bidirectional treatment in gastric cancer PM only in terms of pathological response, having complete or partial response in 60% of patients. Safety was evaluated in all phase II studies. Serious adverse events (CTCAE grade 3–4) occurred in 4%, with a range between 3.2% and 16% of procedures. Only 1 death within the first 30 postoperative days was reported by Rovers for sepsis of unknown origin. A total of 24 out of the 53 included studies involved patients with PM from various origins. The other 29 papers included populations with PM from one origin or from primary tumors that can be assimilated in terms of natural history and prognosis (colorectal cancer *n* = 5, gastric cancer *n* = 10, hepatobiliary and pancreatic cancer *n* = 6, gynecological tumor *n* = 7, mesothelioma *n* = 1). The main characteristics and outcomes of the selected studies are reported in Table 3.

### 3.4. Patient Characteristics

In total, 47 of the 53 included studies described the PCI at the first PIPAC. In 25 studies, PCI was reported as a median with a range from 8 to 29, and 22 as a mean value ranging from 8.6 to 20.

Concerning patients’ performance status, almost all studies enrolled patients with ECOG PS between 0–2; only in one paper was the mean ECOG PS at the first PIPAC higher than 2 [44].

In total, 29 of the 53 studies reported the proportion of patients with ascites at the first PIPAC ranging from 18% to 95% among the series.

Most studies (*n* = 49) involved patients who underwent previous systemic chemotherapy, with an overall weighted means of 95%.

Information on concomitant systemic chemotherapy with PIPAC was available in 42 publications, with 46% of patients receiving the combined treatment.

### 3.5. Feasibility

Concerning the non-access rate or inability to perform PIPAC, data were available in 37 out 53 papers, with a pooled rate of 4% of PIPAC procedures performed (range 0–18.8%).

Among the 53 studies selected for the review, 48 reported in detail the number of patients who underwent 1, 2, or 3 or more PIPAC. Overall, 1669 patients underwent at least 1 PIPAC, 1023 had ≥ 2 PIPAC, and 653 underwent ≥ 3 PIPAC. The overall proportion of patients who completed 3 or more cycles of PIPAC was 39%.

**Figure 2:**
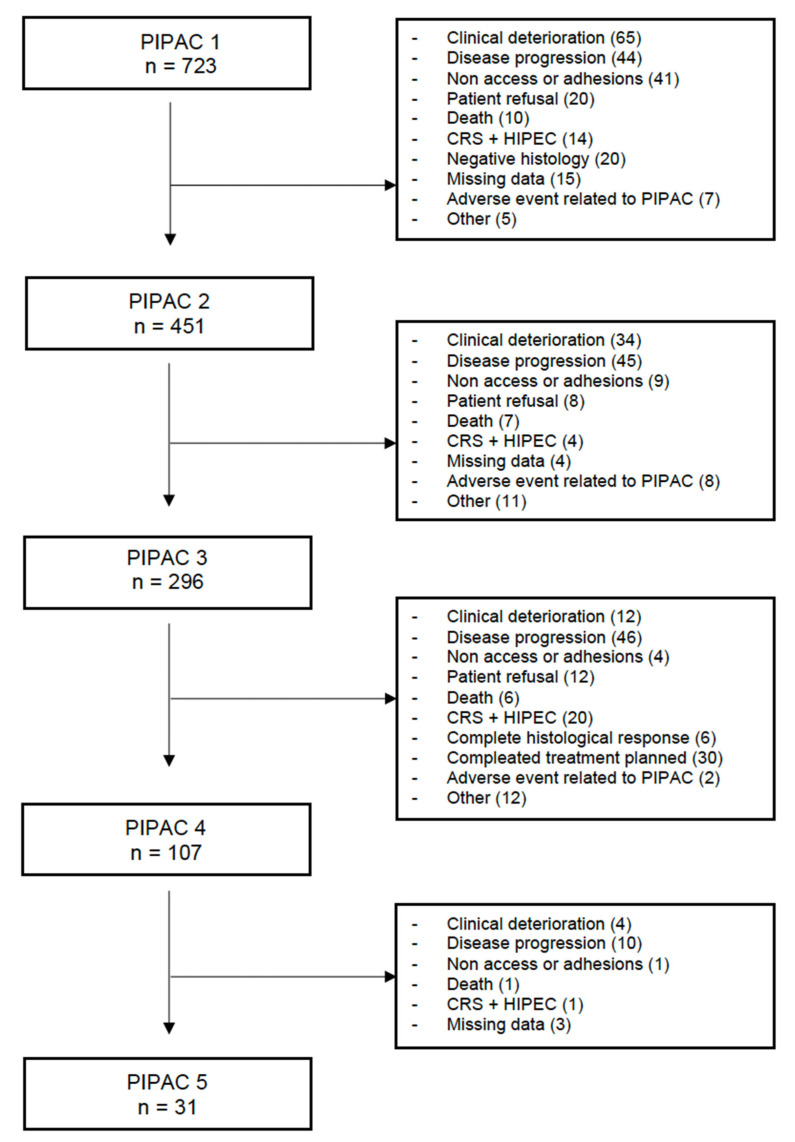
Flow chart of reasons of discontinuation of PIPAC.

Twenty studies reported a summary flowchart of patients interrupting PIPAC courses and the causes of discontinuation. With the limits of the interpretation and the aggregation of data collected, we tried to describe the reasons for the termination of PIPAC planned treatment in the whole population of 723 patients. Clinical deterioration as ileus, bowel obstruction, asthenia, or ascites are the most reported causes of interrupting treatment after the first PIPAC. Disease progression represented the most frequent event for discontinuing treatment after the second and third PIPAC. Non-access to the abdominal cavity/intra-abdominal adhesions was more frequent at the time of the first procedure. PIPAC-related adverse events rarely interrupted the plan of treatment (Figure 2).

**Figure 3:**
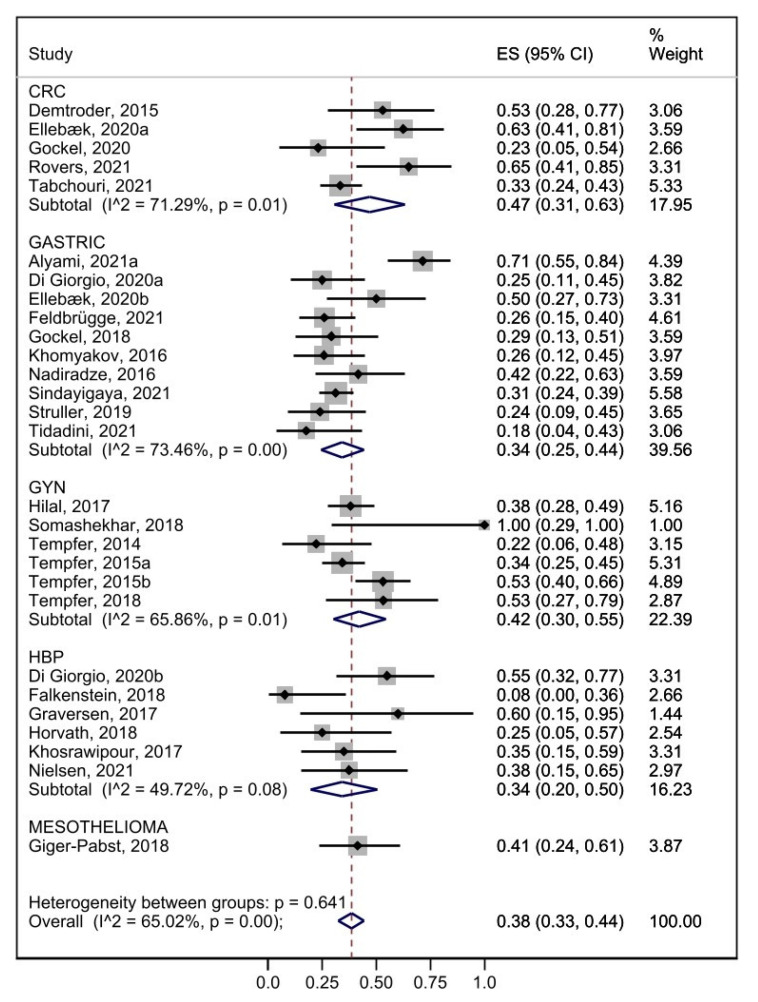
Forest plot of pooled prevalence of PIPAC ≥ 3 stratified by tumor origin [8,10,11,12,13,14,16,17,19,23,24,27,28,32,33,39,43,46,47,48,50,51,52,54,55,57,58,59].

The pooled analysis with the outcome of PIPAC ≥ 3 was also performed per primary tumor, excluding various origins. The proportion of patients affected by peritoneal metastases of colorectal cancer origin who underwent 3 or more PIPAC was 47% (95% CI 0.31–0.63) (I^2^ 71.29% *p* = 0.01), 34% for patients of the gastric one (95% CI 0.25–0.44) (I^2^ 73.46% *p* = 0.00), 42% for gynecological tumors (95% CI 0.30–0.55) (I^2^ 93.1% *p* = 0.00), and 34% for patients with hepatobiliary and pancreatic tumors (95% CI 0.20–0.50) (I^2^ 49.72% *p* = 0.08) (Figure 3).

The outcome of PIPAC ≥ 3 was stratified regarding the extent of peritoneal disease at the first PIPAC by grouping the population with a PCI cut-off of 15. There was no difference in the proportion of patients receiving more than 3 PIPAC between PCI > 15 and PCI ≤ 15, reporting high heterogeneity.

Meta-regression of PIPAC ≥ 3 with previous systemic chemotherapy, concomitant systemic chemotherapy, ECOG PS at first PIPAC, ascites at first PIPAC, and CTCAE 3/4 showed no significant results.

### 3.6. Safety

Data regarding treatment-related adverse events were reported by all studies but four [15,23,33,55]. Severe adverse events considering CTCAE 3–4 were 4% (0% to 38.5%), with the highest rates from phase I dose-escalation trials.

A total of 165 severe adverse events occurred in 1687 patients from studies detailing the type of observed complications.

In details, 46 adverse events, such as leukopenia, anemia, drug allergy, and hepatotoxicity, were secondary to chemotherapy drugs administration (intravenous and intraperitoneal). Twenty-three complications were instead related to the surgical procedure, the most common being bowel perforations and wound infections. Twenty-eight complications related to the natural history of the disease, such as bowel occlusion and ascites, were documented. In total, 29 cases of infective adverse events (pneumonia, urosepsis, and unspecified infections), and 17 leukocytosis whose cause is not specified were also reported. Finally, there were also 29 miscellaneous complications, including abdominal pain, cardiological complications, pulmonary complications, and unspecified.

In total, 50 studies evaluated deaths within the first 30 postoperative days. In the included 1936 patients were registered 26 deaths (1.3%). In the 13 studies that reported deaths, the percentage ranged from 0.5% to 6.8%, and the leading cause of death was disease progression (*n* = 10). In Siebert et al., 2021 were reported six deaths, but the cause was not specified. In three deaths due to heart failure, renal failure, and pulmonary embolism the possible relationship with the procedure was not reported. Three deaths from respiratory failure not related with PIPAC were reported. Only four deaths are related, or possibly related, with the procedure due to iatrogenic bowel perforation and aspiration pneumonia, or sepsis.

### 3.7. Efficacy

The efficacy outcomes of the selected studies are reported in Table 4.

Pathological response has been reported in 38 out of the 53 included studies.

The pooled analysis of all the studies reporting a pathological response was 62% (95% CI 0.54–0.70), with a high degree of heterogeneity (I^2^ 67.99%, *p* = 0.00), but considering only studies with single tumor origin, the pathological response was 68% (95% CI 0.61–0.73) with a limited heterogeneity (I^2^ 28.41%, *p* = 0.09) was documented.

**Figure 4:**
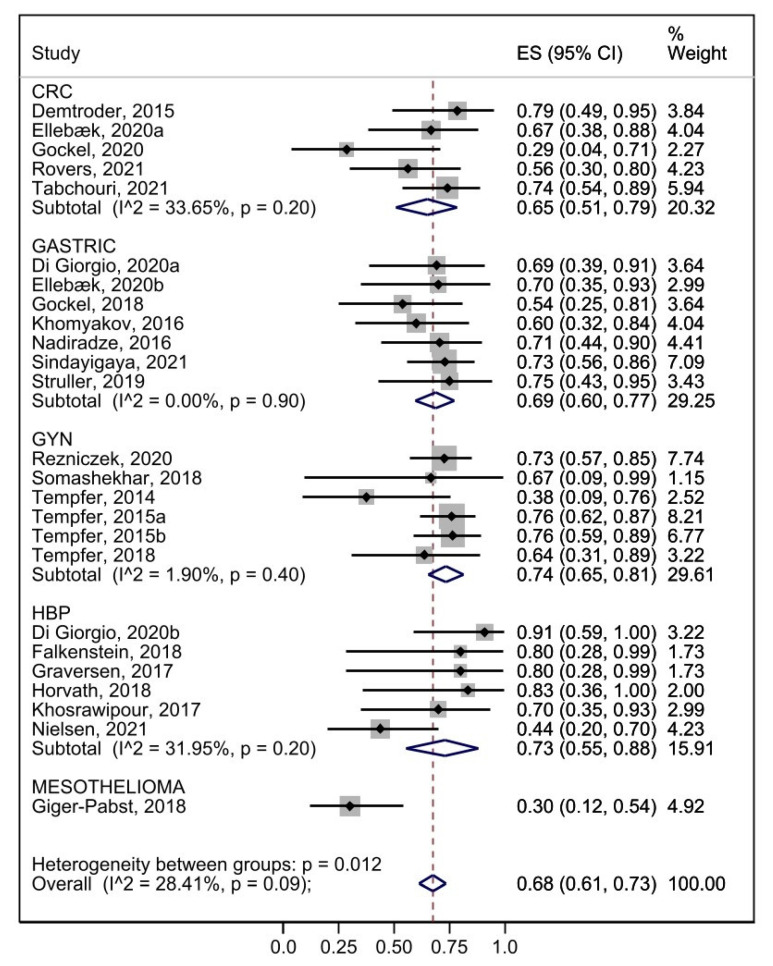
Forest plot of pooled prevalence of histological response stratified by tumor origin [10,11,12,14,16,17,19,24,27,28,32,33,36,39,43,46,47,48,50,51,52,54,57,58,59].

In detail, pathological response was documented in 65% of colorectal cancer patients (95% CI 0.51–0.74) (I^2^ 71.31%, *p* = 0.01), 69% of gastric cancer patients (95% CI 0.60–0.77) (I^2^ 0.00%, *p* = 0.90), 74% of the gynecological tumors (95% CI 0.65–0.81) (I^2^ 1.90%, *p* = 0.40), and 73% of the hepatobiliary and pancreatic patients (95% CI 0.55–0.88) (I^2^ 31.95%, *p* = 0.20) (Figure 4).

Meta-regression of histological response with previous systemic chemotherapy, concomitant systemic chemotherapy, ECOG PS at first PIPAC, ascites at first PIPAC, and CTCAE 3/4 showed no significant results.

Only 10 papers reported data regarding the radiological response to PIPAC using RECIST criteria v. 1.1. In these studies, objective response has a very high variability rate, ranging from 0% to 77.8%. The pooled analysis of the radiological response reported a weighted means of 15% with heterogeneity (I^2^ 79.31%, *p* = 0.0).

PCI variations were reported in fourteen 14 studies as median or mean values. PCI diminished, increased, or remained stable in 8, 1 and 5 studies, respectively, along PIPAC treatments. Specifically, PCI diminished in the two phase II studies reporting data [39,47]. Pooled analysis of PCI variation documented high heterogeneity.

Ascites median or mean values variation along PIPAC treatment was reported in 7 of 53 studies. Ascites variation in milliliter was reported in 14 studies remaining stable and diminishing in 7 and 7, respectively.

In total, 17 out of 53 studies reported conversions to CRS/HIPEC after PIPAC courses for palliative intent. Patients converted to CRS and HIPEC along with PIPAC treatment were reported among colorectal (four studies), gastric (five studies), and various origin (eight studies) PM. Overall 102 patients (7.6%) underwent CRS and HIPEC out of 1335 treated with PIPAC for unresectable disease. In total, 21 patients underwent CRS and HIPEC after the first PIPAC, while 81 patients after at least two PIPAC courses with the majority receiving 3 PIPAC.

### 3.8. Survival Analysis

In total, 27 out of 53 studies reported data regarding median or mean survival. Fifteen studies reported survival from the first PIPAC procedure and twelve from PM diagnosis (Table 3).

In order to avoid potential heterogeneity, the analysis was restricted to the studies including only patients affected by PM of a single origin (colorectal cancer *n* = 2, gastric cancer *n* = 3, hepatobiliary and pancreatic cancer *n* = 5, gynecological tumor *n* = 2, mesothelioma *n* = 1) and providing median survival data from the first PIPAC.

The survival rates at 3, 6, 9 and 12 months are shown in Table 5 and the forest plotsre available as Supplementary Material (Figure S1).

In detail, for colorectal cancer, at 12 months from first PIPAC the estimated survival rate is 53% (95% CI 0.35–0.71), 25% (95% CI 0.05–0.51) for gastric cancer, 59% (95% CI 0.51–0.67) for gynecological origin and 37% (95% CI 0.21–0.54) for the hepatobiliary and pancreatic ones.

All these analyses are burdened by a high heterogeneity. The forest plot of pooled prevalence of survival stratified by tumor origin is available in Supplementary Materials.

Survival was correlated to the number of PIPAC administered in five studies of this review.

Di Giorgio et al. reported better survival for gastric cancer PM patients who repeat PIPAC (9 months for 1 PIPAC group vs. 15 months PIPAC > 1 group). Similarly, De Simone et al. reported median OS for ITT population of 15 vs. 18 months for those patients completing the protocol PP with at least 2 PIPAC. Gockel et al., 2018 reported significantly better survival for those patients with gastric cancer PM able to have more than 3 PIPAC (121 days for 1–2 PIPAC vs. 450 days 3 > PIPAC; *p* = 0.0376). Sindayigaya et al., 2021 documented three or more PIPAC to be an independent prognostic factor for improved OS at multivariate analysis (hazard ratio 0.36; *p* < 0.0001). Tabchouri et al., 2021 documented 3 or more PIPAC as an independent prognostic factor for 12-months survival (OR 4.5; *p* < 0.014) in colorectal cancer PM patients.

## 4. Discussion

In this work, we performed a systematic review and meta-analysis to assess the feasibility, safety, and efficacy of PIPAC. We extensively reported the methodology and results of the statistical meta-analysis of PIPAC patients. Thirteen reviews on PIPAC treatment were published. In their systematic reviews, Grass 2017, Alyami 2019, and Ploug 2020 offered an overview of the topic, stating the non-feasibility of the meta-analysis due to heterogeneity and limited available data [1,65,66]. The present review includes the most recent publications and collects a wider number of patients and data. Being aware of the limitations reported by other authors, we carried out a meta-analysis for the key outcomes of response to PIPAC treatment and subgroup analyses to reduce heterogeneity and provide a more accurate interpretation of the results.

PIPAC is a new treatment modality currently under evaluation. Based on the present review of 4719 procedures in 1990 patients, PIPAC is a feasible, safe procedure that has demonstrated antitumoral activity in terms of pathological regression in about 62% of patients with advanced peritoneal metastasis refractory to standard treatment.

Peritoneal metastases are a common feature of the advanced stages of many intra-abdominal malignancies. It is generally acknowledged that the current treatment options for PM are limited and that outcomes are poor. For unresectable PM, systemic chemotherapy represents the standard of care. However, efficacy is reduced because of weak penetration of agents into peritoneal nodules, with consecutive relative chemoresistance and non-negligible toxicity. In this sense, PM represents a significant area of unmet clinical need and urging for new therapeutic options. PIPAC was developed to treat PM and might fulfill this gap.

There is consistency across studies in terms of the PIPAC technique and the intraperitoneal chemotherapy regimens. As well as describing an almost identical procedural approach, all authors in this review largely use the same chemotherapy during PIPAC. The combination of Cisplatin 7.5 mg/m^2^ + doxorubicin 1.5 mg/m^2^ was used across almost all studies for gastric, ovarian, pancreatic, and peritoneal metastasis, while Oxaliplatin 92 mg/m^2^ for colorectal peritoneal disease. From 2018, after the Tempfer et al. phase I study, expert centers adopted a higher dose of Cisplatin and Doxorubicin (10.5–2.1 mg/m^2^).

Recently, a PIPAC consensus meeting was held in Paris to avoid heterogeneity in treatment protocols. Based on the available literature, in the consensus paper, 90.9% of experts endorsed the higher dose of cisplatin (10 mg/m^2^) + doxorubicin (2.1 mg/m^2^), and 72.7% of experts supported oxaliplatin at 120 mg/m^2^ with a potential reduction to 90 mg/m^2^ in frail patients. Mitomycin C and Nabpaclitaxel were favored as alternative regimens in the framework of controlled trials [67].

This study confirms the feasibility of PIPAC in patients with PM. In fact, less than 4% of patients had non-access to the abdominal cavity during PIPAC or an impossibility to complete the PIPAC procedure. Of note, all series reported in this systematic review considered both synchronous and metachronous peritoneal metastasis, so that patients included already received primary tumor surgical resection or extended surgery, such as cytoreduction and HIPEC procedures for peritoneal disease. Non-access procedures may be related to postoperative or tumoral adherences impeding the achieving to the abdominal cavity or limiting the intraperitoneal space for chemotherapy administration.

Most non-access cases (70%) occurred at the time of the first PIPAC.

Concerning safety, the pooled severe adverse events rate was 4%, ranging from 0 to 38.5%. In total, 26 (1.3%) deaths within the first 30 postoperative days occurred in 1936 patients from studies that evaluated this outcome, with disease progression as the leading cause of death.

Indeed, the higher rates of morbidity and mortality were documented by early series, including very high-risk patients with end-stage disease [32,51]. Recent literature reported severe toxicity < 15% and mortality < 5%, indeed. Furthermore, some series reported 30-day post-operative mortality, not related to the procedure itself, but to the clinical deterioration of very fragile patients. Indeed, these early mortality cases, due to defeated patients’ general conditions, advocate better patient selection. In this sense, experts agreed on considering a life expectancy of less than 3 months, a recent history of bowel occlusion, an inability to oral feed, or the need for parenteral nutrition, and PS ECOG > 3 as contraindications to PIPAC.

PIPAC tolerance was out of the objectives of the present research. However, according to the 2019 review of Alyami et al. on 1810 PIPAC for various indications, mild adverse events were infrequent (CTCAE grade 2 of 12–15%), and PIPAC resulted in a well-accepted procedure.

Treatment response was assessed by several prospective and retrospective studies and was the primary endpoint of all phase II trials. What comes to our attention is that responses differ considerably among studies and when comparing RECIST, PCI, and pathological regression outcomes.

The RECIST criteria, which are the current international standard for solid tumors response assessment, have been used in 4 out of 5 phase II PIPAC trials, showing an objective response rate weighted means of 15% (0–77%). The high variability of radiological responses to PIPAC treatment reported here raises the concept of the inadequacy of RECIST criteria for the evaluation of peritoneal metastasis due to the frequent absence of target lesions to be followed along treatment and the limit to detect micronodular diffuse peritoneal disease. The prognostic role of RECIST criteria for peritoneal disease assessment has already been questioned in the setting of neoadjuvant systemic chemotherapy for patient candidates for cytoreduction surgery. In the post-hoc analysis on the radiological evaluation of a large randomized controlled trial of ovarian cancer PM patients, the RECIST criteria did not predict the progression-free survival of patients undergoing interval debulking surgery [68].

The Peritoneal Cancer Index (PCI) is widely used for preoperative assessment of peritoneal disease extent, and it has been considered as an alternative method of chemotherapy response. Visual PCI was reported at the time of the first PIPAC in all studies examined, but it was rarely documented along the repeated cycles as a response outcome. Only two phase II PIPAC trials reported PCI variations during treatment, documenting a reduction in both studies. Alyami et al., 2017, in a multicenter cohort of patients, documented improvement of PCI in more than half of the patients. However, the visual extent of peritoneal disease represents an operator-dependent procedure and, in patients receiving chemotherapy, may not correspond to the real tumor nodule’s vitality. For these limitations, in our opinion, the role of visual PCI alone has currently limited use as part of multimodal treatment response assessment and needs to be better investigated in future studies.

Pathological response based on peritoneal biopsy was the most prevalent outcome across the studies retrieved. When carried out for single tumor origin, the meta-analysis for pathological regression demonstrated fairly homogenous data (I^2^ 28.41%, *p* = 0.09) among studies and a prevalence of 68%. Pathological regression seems the most reliable outcome in estimating the cytotoxic activity of PIPAC.

Indeed, several critical issues on pathological response emerged from the literature review. First, several methods have been used to assess pathological regression. Secondly, variation exists among studies in the method of assessing tumors, reporting this outcome on the PerProtocol (PP) or IntentionToTreat (ITT) populations, resulting in possible bias. In fact, only a proportion of patients who underwent more than >1 PIPAC are valuable in each study, and the histological response rates do not represent the entire study population. In addition, there is variation in the time of response assessment in relation to the number of PIPAC cycles given. Furthermore, almost one third of patients underwent combined treatment, with a possible added benefit of the concomitant systemic chemotherapy that could not be evaluated separately.

Until today, the response outcomes of pathological, radiological, and macroscopic evaluation have not been correlated to long-term and survival outcomes. In the present review, we attempted a survival analysis of existing data, which suggests even better figures as compared to current therapies. For colorectal PM, the best median survival by systemic chemotherapy was estimated to be 16.3 months (IQR 12.9–19.2) [69]. In this review, among studies focused on colorectal peritoneal metastasis, 2 of them reported a median survival from the first PIPAC of 15.7 and 10.1 months, while the remaining three studies reported median survival from diagnosis of PM with a wide range from 8 to 37.6 months. These data, together with the estimated 12-month survival rate of 53% (95% CI 0.35–0.71), represent an encouraging result for colorectal PM patients with advanced-stage disease, refractory to therapy.

Eight out of ten studies evaluated the survival of gastric cancer PM patients after PIPAC. Median survival was reported from the first PIPAC in 3 studies, ranging widely from 4 to 12.3 months, and in five studies from diagnosis of PM, ranging from 6.7 to 19.1 months. The estimated 12-month survival rate was 25% (95% CI 0.05–0.51) from the first PIPAC. These survival data must be compared to the reported median survival of patients treated with systemic chemotherapy that does not exceed 10.7 months (95% CI 9.1–12.8) [70]. Among patients with recurrent platinum-resistant ovarian cancer, a median survival of 16.6 months is the best outcome reported for systemic chemotherapy [71]. PIPAC literature reported a median survival of 11.0-14.0 months with PIPAC. Impressively, the estimated 12-month survival rate for pancreatic and hepatobiliary PM was 37%. This high rate, as compared to standard treatment survival, may also depend on the strict selection of pancreatic cancer patients with PM who get to PIPAC treatment, but it surely encourages further focused clinical trials.

There are some data in recent literature to support an increase in survival with the amount of PIPAC delivered. In a multicenter cohort study on 586 patients with gastric cancer PM, Alyami et al. reported median survival of 15.4 months from diagnosis and 20.1 months for patients with >3 PIPAC [72]. Balmer et al. reported a longer overall median survival in patients with more than 3 PIPAC compared to PIPAC < 3 patient group (16 vs. 7.2 months *p* < 0.001) in a population of 183 patients with PM from various origins [73]. Furthermore, some authors defined per-protocol treatment as the patient’s completion of three or more cycles of PIPAC. This systematic review reports that the rate of 3 PIPAC range from 0% to 100% across evaluable studies, achieving the rate of 100% in a study with only 3 patients [46]. The pooled analysis shows a PIPAC ≥3 prevalence of 39% (95% CI 0.34–0.45) in the entire population and 47% of colorectal, 34% gastric, 34% HBP, and 42% of ovarian cancer. Although the limitations are due to the wide heterogeneity found, PIPAC ≥ 3 prevalence of 39% expresses that the majority of patients enrolled are not able to complete the planned 3 PIPAC.

In this meta-regression, receiving more than 3 PIPAC does not correlate to the disease extent, considering an arbitrary PCI cut-off of 15. Moreover, the high degree of heterogeneity did not allow us to assess its correlation to the number of previous systemic chemotherapy lines or receiving concomitant systemic chemotherapy.

This systematic review documented a consistent number of patients who dropped out of the PIPAC protocol, with 618 patients over 1669 (37%) receiving only 1 PIPAC. These data have to be interpreted as the results of the learning curve of the centers implementing the procedure who, in an initial phase, experimented with PIPAC in advanced palliative situations. The main reasons for discontinuation of PIPAC treatment according to retrieved articles are disease progression and patient clinical deterioration, followed by patient wish and other medical reasons. PIPAC post-operative complications represent a rare cause of interrupting PIPAC courses.

If the application of a single course of PIPAC is questionable and may result in ineffectiveness, a rigorous patient selection seems to be mandatory. Further research on the prognostic factors for performing multiple PIPAC cycles may improve our current selection performances.

It could be postulated that bringing forward the beginning of PIPAC treatment along the natural history of the peritoneal disease may allow multiple cycles of PIPAC with better survival. Of course, early application of PIPAC in the treatment pathway of PM, such as in the first-line setting, prior to the development of resistance to systemic chemotherapy, will find patients fitter for the combined treatment and able to complete all planned PIPAC treatment.

This research showed emerging data on the combined use of PIPAC with courses of systemic chemotherapy. In total, 42 studies in this review reported the combined treatment, with a range from 0% to 100% of patients. The pooled analysis documented 46% of patients received a bidirectional approach. Although the first experiences evaluated PIPAC as monotherapy, today, most of the expert centers used administer PIPAC along with systemic treatment. The combination of treatments seems de facto feasible, but its safety has to be elucidated in specifically designed phase II studies.

A recent review investigating the concomitant use of systemic chemotherapy and PIPAC failed to provide any conclusions on survival and quality of life in comparison to PIPAC alone [65].

Growing literature on the role of PIPAC as a neoadjuvant treatment to curative surgery is emerging. Two studies fully focused on this topic [15,20]. Overall, the conversion to secondary CRS and HIPEC after PIPAC cycles were documented in 17 studies, with an overall proportion of 7.6%. Although patients converted after the first PIPAC probably already had a low PCI, and PIPAC might not have any influence on the choice of cytoreductive surgery, most patients became candidates for curative resection based on pathological tumor regression or reduction of peritoneal disease extent after repeated PIPAC cycles.

These initial experiences from the retrospective series suggest that strictly selected patients with unresectable peritoneal metastases could be eligible for secondary CRS and HIPEC after repeated PIPAC sessions with palliative intent.

Literature available on PIPAC currently comprises retrospective, prospective papers mainly focused on the feasibility and safety of the procedure, phase I studies reporting dose-escalation, and phase II trials assessing the treatment response to PIPAC as efficacy outcomes. While awaiting more robust evidence from multicenter cohort studies and randomized trials, we designed the present review and meta-analysis to update the results on PIPAC efficacy and provide cumulative preliminary survival outcomes.

This is the first published systematic review that extensively reports and describes the results of the meta-analyses on the use of PIPAC for the treatment of peritoneal metastasis. We are aware of the limitations of the analysis. Most studies included are descriptive non-controlled series lacking predefined endpoints, which largely hampers the reliability of their aggregate analysis. Difficulties in the extraction and aggregation process of inhomogeneous records may reflect the high heterogeneity from the meta-analysis [74]. Concerning phase II evidence, efficacy outcomes vary sensibly, depending on the assessment method, pathological, radiological or clinical, each having its own advantages and drawbacks.

With these assumptions, we tried to summarize the PIPAC state of art.

The selection of patients starting the PIPAC program is crucial to avoid ineffective treatment and to reduce dropouts. Earlier PIPAC application in the disease’s natural history may be worthwhile, as more patients may complete a full PIPAC course. Three or more PIPAC applications, in accordance with the concept of multiple antiblastic administrations, should provide the best survival outcome. Finally, a combination of PIPAC with systemic chemotherapy is practiced and feasible in several centers.

This meta-analysis may serve as a reliable basis for further research. Several ongoing phase II and phase III randomized trials are now evaluating PIPAC, also in the first-line, adjuvant, or neoadjuvant settings. Immediate future directives are completing phase I studies for paclitaxel, irinotecan, and mitomycin C; phase I-II studies for dose-escalation, safety, and efficacy evaluation of the bidirectional treatments; and phase III studies to elucidate quality of life and survival compared to the standard of treatment.

## 5. Conclusions

In conclusion, on the evidence of the present analysis, PIPAC may be a useful treatment option for selected patients with PM with acceptable grade 3 and 4 toxicity and promising survival benefit. Meta-analysis showed the lack of uniformity of data reporting among up-to-date available studies, questioning the reliability of the results. However, in a subset analysis per primary tumor origin, pathological tumor regression was documented in 68% of the studies with acceptable heterogeneity. Pathological regression seems a reliable outcome in estimating PIPAC cytotoxic activity and a potential surrogate endpoint of treatment response.

We recommend uniform selection criteria for patients entering a PIPAC program and highlight the urgent need to standardize items for PIPAC reports and datasets. Consensus on clinical endpoints tailored to PM patients’ clinical settings (QoL. PFS, OS, or date to obstruction) is needed to allow cross-study comparison, homogeneous data, and robust results.

## Figures and Tables

**Table 1:** String search strategy for Medline and Web of Science.

Medline			
Set	Search Terms	Search Type	Results
#1	peritoneal carcinosis OR peritoneal metastases OR peritoneal carcinomatosis	Advanced	35,973
#2	pressurized intraperitoneal aerosol chemotherapy OR pipac	Advanced	278
#3	#1 AND #2	Advanced	191
#4	#3 Filters: from 2011–2021	Advanced	182
**Web of Science**			
**Set**	**Search Terms**	**Search Type**	**Results**
#1	TOPIC: peritoneal carcinosis OR peritoneal metastases OR peritoneal carcinomatosis	Advanced	13,674
#2	TOPIC: pressurized intraperitoneal aerosol chemotherapy or pipac	Advanced	292
#3	#1 AND #2	Advanced	235
#4	#3 Filters: from 2011–2021	Advanced	232

**Table 2:** Phase I dose escalation studies on PIPAC treatment.

Author	Year	Origin	Drugs	PIPAC Dose mg/m^2^
Tempfer	2015	OC	Doxorubicin + Cisplatin	2.1 + 10.5
Dumont	2020	GC, CRC, SBC	Oxaliplatin	90
Kim	2021	GC, CRC, HBPC, AC	Oxaliplatin	120
Robella	2021	GC, OC, PMP	Doxorubicin + Cisplatin	6 + 30
		CRC	Oxaliplatin	135
Celeen	2022 *	GC, CRC, HBPC, AC, OC	Nabpaclitaxel	112.5

OC: ovarian cancer, GC: gastric cancer, CRC: colorectal cancer, SBC: small bowel cancer, HBPC: hepatobiliary-pancreatic cancer, AP: appendix cancer, PMP: pseudomyxoma peritonei. * Study published after the search period.

**Table 3:** Main characteristics of included studies.

**First Author,** **Year of Publication**	**Study Design**	**Primary Tumor**	**Sample Size**	**Number of PIPAC**	ECOG at 1st PIPAC *	PCI at 1st PIPAC *	PCI Post *	**Ascites at 1st PIPAC (%)**	**Previous Systemic Chemotherapy (%)**	**Concomitant Systemic Chemotherapy (%)**	**Non-Access (%)**	**≥3 PIPAC (%)**	**CTCAE 3–4 (%)**	**CTCAE 5 (%)**
Demtroder, 2015	Retrospective	CRC	17	48	1.00	16.0		18.0	94.1	64.7	0.0	52.9	8.3	0.0
Ellebæk, 2020a	Retrospective	CRC	24	74	0.83	10.7 ^§^		29.0	91.7	12.5		62.5	2.7	0.0
Gockel, 2020	Prospective	CRC	13	26	1.00 ^§^	14.0 ^§^		23.0	92.3	38.5	18.8	23.1	0.0	0.0
Rovers, 2021	Phase II	CRC	20	59	0.75	29.0 ^§^			60.0	0.0	6.5	65.0	22.0	5.0
Tabchouri, 2021	Prospective	CRC	102	185		13.0 ^§^	19.0 ^§^	42.5	97.1	56.9	16.4	33.3	3.8	0.5
**Subtotal, sum**			**176**	**392**										
**Subtotal, weighted means**					**NA**	**NA**	**NA**	**ND**	**90**	**30**	**8**	**47**	**6**	**ND**
Alyami, 2021a	Prospective	GASTRIC	42	163		17.0 ^§^			100	100		71.4	3.1	4.7
Di Giorgio, 2020a	Prospective	GASTRIC	28	46	1.00	20.0 ^§^	20.6	38.4	92.9	92.9	4.2	25.0	4.3	0.0
Ellebæk, 2020b	Prospective	GASTRIC	20	52	0.90	13.0		57.0	100			50.0	1.9	0.0
Feldbrügge, 2021	Retrospective	GASTRIC	50	90	0.78	19.0 ^§^			100	100	4.4	26.0	5.6	0.0
Gockel, 2018	Prospective	GASTRIC	24	46	1.00 ^§^	14.0 ^§^			83.3	41.7	9.6	29.2	0.0	0.0
Khomyakov, 2016	Phase II	GASTRIC	31	56		16.0 ^§^		38.7	22.6	100	0.0	25.8	1.8	0.0
Nadiradze, 2016	Retrospective	GASTRIC	24	60		16.0			79.2	33.3	6.3	41.7	11.7	4.2
Sindayigaya, 2021	Prospective	GASTRIC	144	296		15.0 ^§^	15.0 ^§^	50.3	91.0	23.6	6.5	31.3	2.4	1.4
Struller, 2019	Phase II	GASTRIC	25	43	1.00	15.3	12.2 ^§^		100	0.0	2.3	24.0	7.0	0.0
Tidadini, 2021	Retrospective	GASTRIC	17	42	1.00	18.0			100	100	0.0	17.6		
**Subtotal, sum**			**405**	**894**										
**Subtotal, weighted means**					**NA**	**NA**	**NA**	**ND**	**92**	**73**	**4**	**34**	**3**	**ND**
Hilal, 2017	Prospective	GYN	84			18.9			100	7.1		38.1		0.0
Rezniczek, 2020	Retrospective	GYN	44	150	0.70					68.2	4.7		8.0	0.0
Somashekhar, 2018	Prospective	GYN	3	9	2.00 ^§^	19.6		66.6	100			100	0.0	0.0
Tempfer, 2014	Case series	GYN	18	34		17.3		76.2	100		8.1	22.2	14.7	0.0
Tempfer, 2015a	Retrospective	GYN	99	252	1.00 ^§^	16.6		45.0	100		6.7	34.3	7.9	0.0
Tempfer, 2015b	Phase II	GYN	64	130	0.43	16.3		42.0	82.8	0.0	7.8	53.1	6.9	0.0
Tempfer, 2018	Phase I	GYN	15	34	1.00	16.3	14.9	20.0	100	0.0	5.9	53.3	2.9	0.0
**Subtotal, sum**			**327**	**609**										
**Subtotal, weighted means**					**NA**	**NA**	**NA**	**ND**	**100**	**11**	**6**	**42**	**7**	**ND**
Di Giorgio, 2020b	Retrospective	HBP	20	45	1.15	17.0 ^§^		40.0	100	55.0	0.0	55.0	0.0	0.0
Falkenstein, 2018	Prospective	HBP	13	17	1.57	20.0	11.4		53.8	23.1	10.5	7.7	0.0	0.0
Graversen, 2017	Prospective	HBP	5	16	0.60			20.0	100	20.0	0.0	60.0	0.0	0.0
Horvath, 2018	Prospective	HBP	12	23	1.18	10.2	7.0 ^§^	25.0	83.3		0.0	25.0	0.0	0.0
Khosrawipour, 2017	Prospective	HBP	20	41	0.70	15.2	14.9	80.0	100		7.3	35.0	0.0	0.0
Nielsen, 2021	Retrospective	HBP	16		0.68				100	37.5		37.5		
**Subtotal, sum**			**86**	**142**										
**Subtotal, weighted means**					**NA**	**NA**	**NA**	**ND**	**95**	**37**	**2**	**34**	**0**	**ND**
Giger-Pabst, 2018	Retrospective	MESOTHELIOMA	29	74	0.70	19.1 ^§^		95.0	72.4	24.1	12.3	41.4	4.1	3.4
**Subtotal, sum**			**29**	**74**										
**Subtotal, weighted means**					**NA**	**NA**	**NA**	**ND**	**72**	**24**	**12**	**41**	**4**	**ND**
Alyami, 2017	Prospective	VARIOUS	73	164		19.0 ^§^	15.0 ^§^	47.9	100	87.7	3.0	42.5	9.8	6.8
Alyami, 2021b	Prospective	VARIOUS	26	437		16.0 ^§^			100	100			0.0	0.0
Ceribelli, 2020	Prospective	VARIOUS	43	71					100	11.6	6.6	25.6	1.4	0.0
Cuadrado Ayuso, 2021	Prospective	VARIOUS	5	9		27.6 ^§^	27.5	55.0	100	100	0.0	40.0	0.0	0.0
De Simone, 2020	Phase II	VARIOUS	40	100	0.60				72.5	50.0	1.6	50.0	3.0	0.0
Dumont, 2020	Phase I	VARIOUS	10	33		22.0 ^§^	16.5 ^§^		100	20.0			27.3	0.0
Girshally, 2016	Prospective	VARIOUS	21	12		11.5						42.9		
Graversen, 2018a	Prospective	VARIOUS	41	106					90.2	19.5		48.8	0.9	0.9
Graversen, 2018b	Prospective	VARIOUS	35	129		14.1		37.1	91.4	14.3	0.0	77.1	3.9	0.0
Graversen, 2019	Prospective	VARIOUS	33	65		8.6			97.0	36.4	4.6	36.4	0.0	0.0
Hubner, 2017	Retrospective	VARIOUS	42	91	0.86	10.0 ^§^			95.2	2.4	3.2	42.9	1.1	0.0
Katdare, 2019	Retrospective	VARIOUS	16	17	0.76	25.1 ^§^		76.4	81.3	12.5	10.5	0.0	11.8	5.8
Kim, 2021	Phase I	VARIOUS	16	24	0.87	17.0 ^§^	12.0 ^§^	68.8	100		4.0	0.0	4.2	0.0
Kurtz, 2018	Prospective	VARIOUS	71	142		19.3		35.2	84.5	59.2	7.7	28.2	3.5	1.4
Odendahl, 2015	Retrospective	VARIOUS	91	158	1.00	16.0			85.7				5.7	2.0
Račkauskas, 2021	Retrospective	VARIOUS	15	34		8.0 ^§^	5.0 ^§^	70.0	100			53.3	8.8	0.0
Robella, 2016	Prospective	VARIOUS	14	40		17.0 ^§^			100	92.9	0.0	71.4	0.0	0.0
Robella, 2021	Phase I-II	VARIOUS	13	13	0.70	14.0 ^§^		38.0		0.0		0.0	38.5	0.0
Sgarbura, 2019	Retrospective	VARIOUS	101	251		19.0 ^§^		46.0	92.1	46.5	3.2	47.5	6.4	1.0
Siebert, 2021	Prospective	VARIOUS	134	397		18.0				100			3.5	3.5
Solass, 2014	Case series	VARIOUS	3	12	3.00	12.0			100	0.0		66.7	8.3	0.0
Somashekhar, 2019	Prospective	VARIOUS	7	21	0.92	17.4		27.0	100			100	0.0	0.0
Taibi, 2021	Retrospective	VARIOUS	69	147		16.3 ^§^	16.0 ^§^		100	78.3	0.0	31.9	7.5	0.0
Willaert, 2019	Prospective	VARIOUS	48	135		21.2 ^§^		37.5	89.6	58.3	2.2	58.3	12.6	0.0
**Subtotal, sum**			**967**	**2608**										
**Subtotal, weighted means**					**NA**	**NA**	**NA**	**ND**	**96**	**48**	**2**	**40**	**4**	**ND**
**Total, sum**			**1990**	**4719**										
**Total, weighted means**														
**with various**					**NA**	**NA**	**NA**	**ND**	**95**	**46**	**4**	**39**	**4**	**ND**
**without various**					**NA**	**NA**	**NA**	**ND**	**94**	**44**	**5**	**38**	**4**	**ND**

* mean or median; § median. Abbreviations: NA = not applicable; ND = not disponible.

**Table 4:** Efficacy outcomes.

**First Author,** **Year of Publication**	**Primary Tumor**	**Histological Response (%)**	**Radiological Response (%)**	**Overall Survival (Median)**
Demtroder, 2015	CRC	78.6		15.7 ^#^
Ellebæk, 2020a	CRC	66.7		37.6
Gockel, 2020	CRC	28.6		10.1 ^#^
Rovers, 2021	CRC	56.3	0.0	8.0
Tabchouri, 2021	CRC	74.1		13.0
**Subtotal, weighted means**		**65**	**0**	**NA**
Alyami, 2021a	GASTRIC			19.1
Di Giorgio, 2020a	GASTRIC	69.2		12.3 ^#^
Ellebæk, 2020b	GASTRIC	70.0		4.7 ^#^
Feldbrügge, 2021	GASTRIC			
Gockel, 2018	GASTRIC	53.8		4.0 ^#^
Khomyakov, 2016	GASTRIC	60.0		
Nadiradze, 2016	GASTRIC	70.6		15.4
Sindayigaya, 2021	GASTRIC	73.0		11.0
Struller, 2019	GASTRIC	75.0	12.0	6.7
Tidadini, 2021	GASTRIC			12.8
**Subtotal, weighted means**		**69**	**12**	**NA**
Hilal, 2017	GYN			
Rezniczek, 2020	GYN	72.7	BC 42.9, EC 25.0	19.6 ^#^
Somashekhar, 2018	GYN	66.7		
Tempfer, 2014	GYN	37.5		
Tempfer, 2015a	GYN	76.0		14.1 ^#^
Tempfer, 2015b	GYN	76.5	3.2	
Tempfer, 2018	GYN	63.6		
**Subtotal, weighted means**		**74**	**21**	**NA**
Di Giorgio, 2020b	HBP	90.9		10.3 ^#^
Falkenstein, 2018	HBP	80.0		2.8 ^#^
Graversen, 2017	HBP	80.0	0.0	14.0
Horvath, 2018	HBP	83.3		13.9 ^#^
Khosrawipour, 2017	HBP	70.0		8.4 ^#^
Nielsen, 2021	HBP	43.8		9.9 ^#^
**Subtotal, weighted means**		**73**	**0**	**NA**
Giger-Pabst, 2018	MESOTHELIOMA	30.0		26.6 ^#^
**Subtotal, weighted means**		**30**	**-**	**NA**
Alyami, 2017	VARIOUS			
Alyami, 2021b	VARIOUS			
Ceribelli, 2020	VARIOUS	42.9		
Cuadrado Ayuso, 2021	VARIOUS	50.0		
De Simone, 2020	VARIOUS	17.9	17.5	18.1
Dumont, 2020	VARIOUS	20.0		
Girshally, 2016	VARIOUS	100	77.8	
Graversen, 2018a	VARIOUS			
Graversen, 2018b	VARIOUS	66.7		
Graversen, 2019	VARIOUS	60.0		
Hubner, 2017	VARIOUS			
Katdare, 2019	VARIOUS			
Kim, 2021	VARIOUS	66.7	0.0	
Kurtz, 2018	VARIOUS	25.6		11.8 ^#^
Odendahl, 2015	VARIOUS			
Račkauskas, 2021	VARIOUS			25.0 ^#^
Robella, 2016	VARIOUS		35.7	
Robella, 2021	VARIOUS		0.0	
Sgarbura, 2019	VARIOUS			102.0
Siebert, 2021	VARIOUS			
Solass, 2014	VARIOUS	100		6.2
Somashekhar, 2019	VARIOUS	57.1		
Taibi, 2021	VARIOUS	53.8		
Willaert, 2019	VARIOUS			
**Subtotal, weighted means**		**53**	**20**	**NA**
**Total, weighted means**				
**with various**		**62**	**15**	**NA**
**without various**		**68**	**11**	**NA**

#: from 1st PIPAC. Abbreviations: NA = not applicable.

**Table 5:** Estimated survival at 3, 6, 9, 12 months.

**Survival at:**	**3 Months**	**6 Months**	**9 Months**	**12 Months**
	ES (95% CI)	ES (95% CI)	ES (95% CI)	ES (95% CI)
**CRC**	**0.87 (0.71–0.97)**	**0.73 (0.56–0.88)**	**0.60 (0.41–0.77)**	**0.53 (0.35–0.71)**
Demtroder, 2015	0.88 (0.64–0.99)	0.76 (0.50–0.93)	0.65 (0.38–0.86)	0.59 (0.33–0.82)
Gockel, 2020	0.85 (0.55–0.98)	0.69 (0.39–0.91)	0.54 (0.25–0.81)	0.46 (0.19–0.75)
**GASTRIC**	**0.71 (0.52–0.87)**	**0.49 (0.25–0.73)**	**0.35 (0.12–0.62)**	**0.25 (0.05–0.51)**
Di Giorgio, 2020a	0.86 (0.67–0.96)	0.71 (0.51–0.87)	0.61 (0.41–0.78)	0.50 (0.31–0.69)
Ellebæk, 2020b	0.65 (0.41–0.85)	0.40 (0.19–0.64)	0.25 (0.09–0.49)	0.15 (0.03–0.38)
Gockel, 2018	0.58 (0.37–0.78)	0.33 (0.16–0.55)	0.21 (0.07–0.42)	0.13 (0.03–0.32)
**GYN**	**0.88 (0.82–0.93)**	**0.77 (0.70–0.84)**	**0.67 (0.59–0.75)**	**0.59 (0.51–0.67)**
Rezniczek, 2020	0.91 (0.78–0.97)	0.82 (0.67–0.92)	0.73 (0.57–0.85)	0.66 (0.50–0.80)
Tempfer, 2015a	0.86 (0.77–0.92)	0.75 (0.65–0.83)	0.65 (0.54–0.74)	0.56 (0.45–0.66)
**HBP**	**0.76 (0.63–0.87)**	**0.59 (0.42–0.75)**	**0.46 (0.27–0.66)**	**0.37 (0.21–0.54)**
Di Giorgio, 2020b	0.80 (0.56–0.94)	0.65 (0.41–0.85)	0.55 (0.32–0.77)	0.45 (0.23–0.68)
Falkenstein, 2018	0.46 (0.19–0.75)	0.23 (0.05–0.54)	0.08 (0.00–0.36)	0.08 (0.00–0.36)
Horvath, 2018	0.83 (0.52–0.98)	0.75 (0.43–0.95)	0.67 (0.35–0.90)	0.58 (0.28–0.85)
Khosrawipour, 2017	0.80 (0.56–0.94)	0.60 (0.36–0.81)	0.50 (0.27–0.73)	0.35 (0.15–0.59)
Nielsen, 2021	0.81 (0.54–0.96)	0.69 (0.41–0.89)	0.56 (0.30–0.80)	0.44 (0.20–0.70)
**MESOTELIOMA**	-	-	-	-
Giger-Pabst, 2018	0.93 (0.77–0.99)	0.86 (0.68–0.96)	0.79 (0.60–0.92)	0.72 (0.53–0.87)

## Supplementary Materials

The following supporting information can be downloaded at: https://www.mdpi.com/article/10.3390/cancers15041125/s1, Table S1: PRISMA checklist; Table S2: Study quality assessment according to the Newcastle-Ottawa Scale (NOS); Figure S1: Forest plot of pooled prevalence of estimated survival at 3, 6, 9, 12 months.
