# Supplementary material for: 10 Years of Pressurized Intraperitoneal Aerosol Chemotherapy (PIPAC): A Systematic Review and Meta-Analysis
Source: Cancers (Basel). 2023 Feb 9;15(4):1125. doi: 10.3390/cancers15041125 (PMC9954579; doi:10.3390/cancers15041125)
Supplement: Supplementary file 1 [file cancers-15-01125-s001.zip › TABLE S2 - Study quality assessment according to the Newcastle-Ottawa Scale (NOS)..pdf]

Table S2. Study quality assessment according to the Newcastle-Ottawa Scale (NOS).

	Demtroder, 2015	Ellebæk, 2020[a]	Gockel, 2020	Tabchouri, 2021	Rovers, 2021	Nadiraze, 2016	Feldbrügge, 2021	Gockel, 2018	Di Giorgio, 2020[a]	Alyami, 2021[a]	Sindayigaya, 2021
**Group selection**											
1) Representativeness of the exposed cohort	*	*	*	*	*	*	*	*	*	*	*
2) Selection of the non-exposed cohort	-	-	-	*	-	-	*	-	-	*	-
3) Ascertainment of exposure	*	*	*	*	*	*	*	*	*	*	*
4) Demonstration that outcome of interest was not present at start of study	-	*	*	*	*	-	*	-	*	*	*
**Comparability**											
5) Comparability of cohorts on the basis of the design or analysis controlled for confounders	**	*	*	*	-	-	*	*	**	*	*
**Outcome**											
6) Assessment of outcome	*	*	*	*	*	*	*	*	*	*	*
7) Was follow-up long enough for outcomes to occur	*	*	-	*	*	*	-	*	*	*	*
8) Adequacy of follow-up of cohorts	*	-	-	*	*	*	-	*	*	*	*
**Summary score (maximum of 9 stars)**	**7**	**6**	**5**	**8**	**6**	**5**	**6**	**6**	**8**	8	7

	Khomyakov, 2016	Struller, 2019	Ellebæk, 2020[b]	Tidadini, 2021	Tempfer, 2014	Tempfer, 2015[a]	Tempfer, 2015[b]	Tempfer, 2018	Rezniczek, 2020	Hilal, 2017	Somashekhar, 2018	Di Giorgio, 2020b
**Group selection**												
1) Representativeness of the exposed cohort	*	*	*	*	*	*	*	*	*	*	-	*
2) Selection of the non-exposed cohort	-	-	-	*	-	-	-	-	-	*	-	-
3) Ascertainment of exposure	*	*	*	*	*	*	*	*	*	*	*	*
4) Demonstration that outcome of interest was not present at start of study	*	*	*	*	*	*	*	*	-	-	*	*
**Comparability**												
5) Comparability of cohorts on the basis of the design or analysis controlled for confounders	-	*	*	**	*	*	*	*	**	*	*	**
**Outcome**												
6) Assessment of outcome	*	*	*	*	*	*	*	*	*	*	*	*
7) Was follow-up long enough for outcomes to occur	*	*	*	*	*	*	*	*	*	*	*	*
8) Adequacy of follow-up of cohorts	*	*	-	*	*	*	*	*	*	*	*	*
**Summary score (maximum of 9 stars)**	**6**	**7**	**6**	**9**	**7**	**7**	**7**	**7**	**7**	**7**	**6**	**8**

	Nielsen, 2021	Graversen, 2017	Falkenstein, 2018	Horvath, 2018	Khosrawipour, 2017	Giger-Pabst, 2018	Alyami, 2017	Alyami, 2021[b]	Ceribelli, 2020	Cuadrado Ayuso, 2021	De Simone, 2020
**Group selection**											
1) Representativeness of the exposed cohort	*	-	*	*	*	*		*	*		*
2) Selection of the non-exposed cohort	-	-	-	*	-			*			
3) Ascertainment of exposure	*	*	*		*	*	*	*	*	*	*
4) Demonstration that outcome of interest was not present at start of study	-	-	*	*	-		*			*	*
**Comparability**											
5) Comparability of cohorts on the basis of the design or analysis controlled for confounders	*	**	**	*	-	*	*		*	*	**
**Outcome**											
6) Assessment of outcome	*	*	*	*	*	*	*		*	*	*
7) Was follow-up long enough for outcomes to occur	*	-	*	*	*	*		*	*		*
8) Adequacy of follow-up of cohorts	*	-	*	*	*	*		*	*		*
**Summary score (maximum of 9 stars)**	**6**	**4**	**8**	**7**	**5**	**6**	**4**	**5**	**6**	**4**	**8**

	Dumont, 2020	Girshally, 2016	Graversen, 2018[a]	Graversen, 2018[b]	Graversen, 2020	Hubner, 2017	Katdare, 2019	Kim, 2020	Kurtz, 2018	Odendahl, 2015	Račkauskas, 2021
**Group selection**											
1) Representativeness of the exposed cohort	*	*	*	*	*	*		*	*	*	*
2) Selection of the non-exposed cohort		*		*		*				*	-
3) Ascertainment of exposure	*	*	*	*	*	*	*	*	*	*	*
4) Demonstration that outcome of interest was not present at start of study			*	*	*	*		*	*	*	*
**Comparability**											
5) Comparability of cohorts on the basis of the design or analysis controlled for confounders	*	**	*	*	*	*		**	*	*	*
**Outcome**											
6) Assessment of outcome	*	*	*	*	*	*	*	*	*	*	*
7) Was follow-up long enough for outcomes to occur	*	*					*	*	*	*	*
8) Adequacy of follow-up of cohorts	*	*	*	*	*			*	*	*	*
**Summary score (maximum of 9 stars)**	**6**	**8**	**6**	**8**	**6**	**5**	**3**	**8**	**7**	**8**	**7**

	Robella, 2016	Robella, 2021	Sgarbura, 2019	Siebert, 2021	Solass, 2014	Somashekhar, 2019	Taibi, 2021	Willaert, 2019
Group selection								
1) Representativeness of the exposed cohort	*		*	*	-	-	*	*
2) Selection of the non-exposed cohort	-		-	*	-	-		-
3) Ascertainment of exposure	*	*	*	*	*	*	*	*
4) Demonstration that outcome of interest was not present at start of study	*	*	*	*	*	*		*
Comparability								
5) Comparability of cohorts on the basis of the design or analysis controlled for confounders	*	*	*		*	*	**	*
Outcome								
6) Assessment of outcome	*	*	*	*	*	*	*	*
7) Was follow-up long enough for outcomes to occur	*	*	*	*	*	*	*	*
8) Adequacy of follow-up of cohorts	*	*	*	*	*	*	*	*
Summary score (maximum of 9 stars)	7	6	7	7	6	6	7	7
