# Supplementary figures and images for: 10 Years of Pressurized Intraperitoneal Aerosol Chemotherapy (PIPAC): A Systematic Review and Meta-Analysis
Source: Cancers (Basel). 2023 Feb 9;15(4):1125. doi: 10.3390/cancers15041125 (PMC9954579; doi:10.3390/cancers15041125)

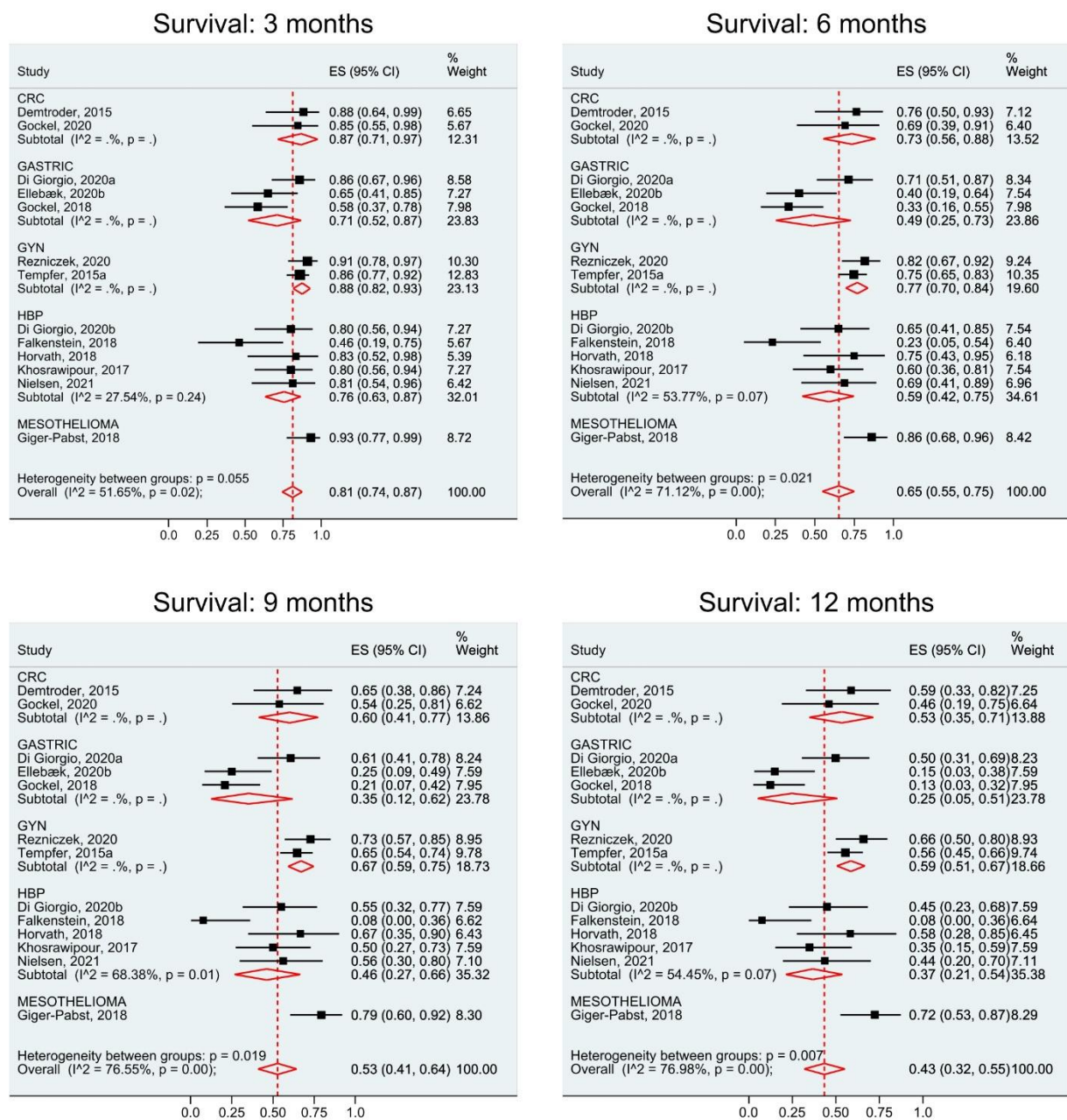

**Figure S1.** Forest plot of pooled prevalence of estimated survival at 3, 6, 9, 12 months.

Supplement: Supplementary file 1 [file cancers-15-01125-s001.zip › Figure S1.Forest plot of pooled prevalence of estimated survival at 3, 6, 9, 12 months..pdf]
